# The effects of cryopreservation on the acrosome structure, enzyme activity, motility, and fertility of bovine, ovine, and goat sperm

**DOI:** 10.1590/1984-3143-ar2020-0219

**Published:** 2021-03-08

**Authors:** Wei Sun, Shan Jiang, Jie Su, Jia Zhang, Xiangnan Bao, Rui Ding, Peixin Shi, Shufang Li, Caixia Wu, Gaoping Zhao, Guifang Cao, Qing-Yuan Sun, Haiquan Yu, Xihe Li

**Affiliations:** 1 College of Life Science and The State Key Laboratory of Reproductive Regulation and Breeding of Grassland Livestock, Inner Mongolia University, Hohhot, P.R. China; 2 Inner Mongolia Saikexing Institute of Breeding and Reproductive Biotechnology in Domestic Animal, Hohhot, China; 3 College of Veterinary Medicine, Inner Mongolia Agricultural University, Hohhot, P.R. China; 4 Institute of Zoology, Chinese Academy of Sciences, Beijing, P.R. China

**Keywords:** bovine, ovine, goat, cryopreservation, sperm acrosome

## Abstract

The study was designed to investigate the effects of cryopreservation on bovine, ovine, and goat sperm motility, acrosome structure, enzyme activity, and fertilization ability. Percentage of sperm with hyaluronidase enzyme (HYD) activity was detected by a modified sodium hyaluronate-gelatin membrane. The N-α-benzoyl-DL-arginine-p-nitroanilide (BNPNA) method was used to assess the sperm acrosome enzyme (ACE). The mean percentage of sperm acrosome integrity dropped significantly (*P* < 0.01) after cryopreservation. The ACE activity of bovine sperm (100.48) was higher (*P* < 0.01) than that of ovine (57.88) or goat sperm (50.30), while the percentage of sperm with HYD activity of bovine (71.10%) and ovine (67.60%) sperm was higher than that of goat sperm (58.52%) after cryopreservation (*P* < 0.01). Sperm motility was positively correlated with the activity of the two acrosome enzymes before and after cryopreservation (*P* < 0.01). Cryopreservation had a negative effect on acrosomal morphology, motility, and acrosomal enzyme activity in their sperm. The fertilization ability of ovine and goat sperm decreased significantly after cryopreservation, but that of frozen bovine sperm did not differ significantly when compared with fresh sperm. There was no significant difference between ovine and goat sperm indices, except for percentage of sperm with HYD activity.

## Introduction

Cryopreservation is the best method for long-term preservation of mammalian sperm used for artificial insemination (AI). However, freeze-thawing procedures may strongly impair the sperm function (Yeste, 2016), Therefore, the enhancement of sperm cryopreservation outcomes remains a major challenge, especially in ruminant animals, which include cattle, sheep, goats and other species (Pulina et al., 2017). To futher improve sperm activity before and after cryopreservation, the ACE and HYD activity were explored in this study, because Sperm motility was positively correlated with ACE and HYD activity. In 1787, the Italian scientist Spallazani froze firstly human and horse sperm at −17°C in snow, discovering a very low survival rate after thawing, which opened the prologue of sperm cryopreservation (He and Woods, 2004). A breakthrough was made with the discovery of the protective effect of glycerol on human and poultry sperm (Polge et al., 1949). The first mammal to be conceived via AI using frozen sperm was a calf in 1951, which frozen bovine spermatozoa were produced by the Ryding Animal Artificial Insemination Research Center (Curry, 2000).

The method of sperm cryopreservation and freezing medium was very vital for cryopreservation outcomes. Different proteins, antioxidants and cryoprotective agents are being incorporated into the freezing medium for increasing sperm cryosurvival. Such improvements, however, have not yet reached the desired level because many sperms still lose their viability after cryopreservation (Hezavehei et al., 2018). As a consequence, changing the protocol of sperm cryopreservation or adding special nutrition into freezing medium were feasible for improving activity, motility, and fertility of bovine, ovine and goat sperm (Ahmadi et al., 2016). During the cryopreservation process in ruminant, spem membrane integrity change, oxidative stress and acrosome damage are different among cattle, goat, deer and other species. Therefore, the improvement of sperm cryopreservation remains an unresolved issue (Peris-Frau et al., 2020). In consequence, if the damage can be reduced during cryopreservation, the survival rate of the sperm will increase after cryopreservation. In some publications, when vitamin and Na_2_SeO_3_ were respectively added to the freezing medium, sperm vitality was significantly increased (Mohamed et al., 2020). Moreover, the rate of early apoptosis, late apoptosis and necrosis were significantly decreased after cryopreservation, therefore, changing constituent of freezing medium maybe improve quality of sperm. In addition, *Moringa oleifera* leaves alcoholic extracts (MOLE) can reduce oxidative stress in rams’ seminal plasma (Shokry et al., 2020).

In order to prevent sperm damage before and after cryopreservation, further experiments are needed to be performed to improve sperm viability and motility during cryopreservation processes. Due to sperm change difference in the domestic species during cryopreservation processes (Purdy, 2006), the objective of this study was to compare the differences in acrosome structure, enzyme activity, sperm motility and fertilization ability of bovine, ovine and goat sperm before and after cryopreservation, providing a theoretical foundation for further improving the quality of frozen sperm in these species.

## Methods

### Reagents

All reagents were purchased from Sigma-Aldrich Co (St. Louis, MO, USA), unless indicated otherwise.

### Semen collection

Five strong-bodied Holstein bulls, five male cashmere goats, and five male Mongolian sheep, under regular feeding management at a station of the Inner Mongolia Saikexing Reproductive Biotechnology (Hohhot, China) Co., Ltd., were used for semen collection. A total of 75 sperm ejaculates from bovines, goats, and ovines (25 ejaculates of each species) were collected by the artificial vagina method. Antibiotics were then added to the semen according to the following dosage: 1 mL semen with 6 µL lincomycin (50 mg/mL), 6 µL tylosin (19 mg/mL), and 8 µL gentamicin (99.01 mg/mL). Subsequently, the semen samples were delivered to the laboratory at 18°C 10 µL of semen was placed on a glass slide and sperm motility was examined using a microscope (Nikon Corporation, Eclipse 80i, Japan). Sperm concentration was determined using a sperm density meter (IMV Technologies, Accucell, France). The ejaculates were evaluated and used for further tests if the following criteria were met: individual motility >70% and total abnormality <10%.

### Sperm freezing and thawing process

Sperm samples were diluted with Tris-citric acid egg yolk glycerol extender (TCEYG, pH 6.8) comprised of Tris (hydroxymethyl) aminomethane (3.53% w/v, Fluka, Buchs, Switzerland), citric acid (1.72% w/v, Fluka), egg yolk (20.00% v/v), fructose (1.26% w/v, Merck, Darmstadt, Germany), glycerol (10.00% v/v, Merck), and antibiotics (benzyl penicillin 1000 IU/mL, Sigma) and streptomycin sulfate (100 µg/mL, Sigma). The final concentration of the frozen sperm was 2.0 × 10^9^ sperm cells/mL. Diluted spermatozoa were cooled to 4°C in 90 min, and packaged into 0.25 mL straws. Sperm straws were frozen by routine procedures (using a controlled-rate freezer) on racks in static liquid nitrogen vapor. After one day in liquid nitrogen, the samples were thawed in a circulating water bath at 37°C for 1 min. Thawed spermatozoa were transferred to a 1.5 mL centrifuge tube and held in the water bath at 37°C throughout the entire experimental process.

### Examination of sperm acrosome structure

#### Sperm acrosome integrity

**Figure 1:**
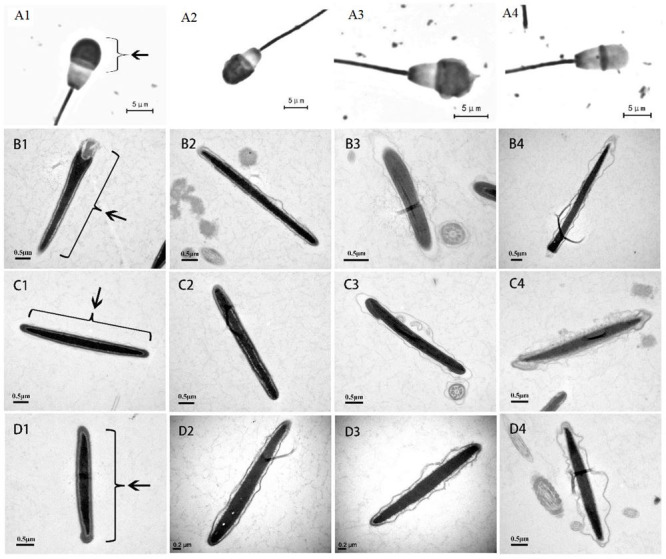
Morphological classification of the sperm acrosome in bovine, ovine and goat. A1-A4: Giemsa staining of bovine sperm head, and B1-B4, C1-C4 and D1-D4: E-microscope analysis in bovine, ovine and goat sperm heads, respectively. A1, B1, C1 and D1: Sperm head with whole acrosome by arrowhead indicated, and A2 to A4, B2 to B4, C2 to C4 and D2 to D4 showing partly and totality lost acrosome structure of sperm head in each species.

Spermatozoa were dried at room temperature for staining with Giemsa. Slide smears were made from samples of spermatozoa and than fixed in neutral formol-saline (5% formaldehyde) for 15 min. The smeared slide were rinsed in tap-water and then stained in the following solution of 3 mL Giemsa, 2 mL Sorensen's buffer (pH 7.0) and 45 mL distilled water. The slide was examined under a microscope (Nikon ECLIPSE 80i), and acrosomal integrity was determined by the morphological of sperm acrosome (Figure 1). At least 200 sperm per slide were examined and repeat three times.

#### Ultrastructure of sperm

The plasma of fresh samples group was removed by centrifugation. Spermatozoa were fixed in 2.5% glutaraldehyde (0.1 M PBS, pH 7.2) for 30min at 4°Cand then mix 2% agar with sperm as 1:1 and set at 4°C. The gel block were fixed in 2.5% glutaraldehyde for 12 h at 4°C, and the samples were rinsed with 0.1 M phosphate buffered saline (PBS, pH 7.2), subsequently fixed in 1% osmium tetroxide for 2 h at room temperature. Alcohol dehydration with a concentration gradient was performed after rinsing with the same PBS, finally treated with pure acetone. Treated samples of spermatozoa with acetone and the same amount of penetration, embedding agent (DDSA+NMA+DBP+ Epon 812). The embedded blocks are aggregated in an incubator. Thin sections were cut showing silver to yellowish colors and were stained with uranyl acetate and lead citrate. Sections were examined and photographed with a Hitachi H-7650 transmission electron microscope. The sperm ultrastructure was classified as follows: acrosome intact (A1, B1, C1, D1), expansion of the plasma membrane with intact acrosome (A2, B2, C2, D2), broken outer acrosome membrane with part of acrosome material leaked (A3, B3, C3, D3), and broken acrosome membrane with most acrosome material lost (A4, B4, C4, D4) (Figure 1), in bovine, ovine and goat respectively.

### Assessment of sperm acrosin activity

#### Assessment of percentage of sperm with HYD activity

The percentage of sperm with HYD activity was detected by modified sodium hyaluronate-gelatin membrane. Spermatozoa were centrifuged at 2400 rpm for 10 min with PBS solution and sediment (sperm) was diluted with PBS solution to obtain 1–2×10^7^ sperm cells/mL. Aliquots (30 µL) of sperm were then used to prepare smears on gelatin membrane and the slides were incubated in a dark and moist chamber for 3 h at 37°C. After air-drying, spermatozoa were observed under the microscope. The sperm positive reaction rate of HYD was assessed, with at least 200 sperm per slide recorded and repeat three times.

#### Assessment of sperm ACE by the BNPNA method

The BNPNA method was used to assess the activity of ACE. Semen was obtained by masturbation after 5-7 days of abstinence. Semen samples were liquefied in a 37°C water bath before analysis. The required amount of semen containing 7.5×10^6^ sperm was placed into a plastic tube, and centrifuged at 1500 rpm for 10 min. The absorbance at 400nm was determined by spectrophotometer 722. One IU of acrosin activity was defined as the substrate amount that hydrolyzed 1.0 µmol/min of BAPNA at 24°C. Formula: Acrosin activity (µIU) /7.5 × 10^6^ sperm = (experimental OD value - control OD value) × 10^6^ /1485 × 7.5 (Cui et al., 2000).

### Assessment of sperm motility

Sperm motility was assessed by microscopic observation and the CASA system on a warm (38°C) stage. Before evaluating motility, sperm samples were adjusted to a concentration of 5.0×10^8^ sperm cells/mL. A 10 µL sample of diluted sperm was placed on a slide and covered with a coverslip (18×18 mm). We examined a minimum of 10 microscopic fields, and sperm motility was expressed as the percentage of motile sperm with moderate to vigorous linear progressive movement in all microscopic fields examined.

### Assessment of sperm in vitro fertilization (IVF) ability

Bovine, ovine, and goat ovaries were obtained from a local slaughterhouse and transported to the laboratory at 35°C in 0.9% NaCl containing 70 mg/mL kanamycin. Cumulus-oocyte complexes (COCs) were aspirated from medium-sized follicles (approximately 5 mm in diameter) with a 10 mL disposable syringe. Only COCs surrounded by a compact cumulus mass with an evenly granulated cytoplasm were harvested, and subsequently washed three times in maturation medium consisting of Tissue Culture Medium 199 (IVM-M199) with Earle's salts, L-glutamine, 26.19 mmol/L sodium bicarbonate, and 25 mmol/L HEPES plus 10% (v:v) fetal bovine serum (Hyclone, Logan, UT, USA), 1% antibiotic/antimycotic (Gibco, Grand Island, NY, USA), 0.8% BSA,0.33 mmol/L pyruvate, 3.3 mmol/L lactate, 1 mmol/L glutamine, 1×MEM essential amino acid, 1× nonessential amino acid and 50 mg /mL gentamicin. About 60 to 80 COCs were transferred into each well of a Nunc 4-well multi-dish containing 500 µL pre-equilibrated maturation medium, previously covered with warm mineral oil. The COCs were cultured for 22 h at 38.5°C in 5% CO_2_ and high humidity.

Spermatozoa were washed twice with BO medium for bovine sperm, SOF medium for ovine and goat sperm and centrifuged at 1500 rpm for 5 min before collecting the supernatant. BO fertilization medium (bovine) and SOF fertilization medium (ovine and goat) were placed separately in 50 µL drops covered with warm mineral oil in a 35 mm culture dish and incubated at 38.5°C in 5% CO_2_. The COCs that matured in vitro were washed three times and placed in 50 µL drops of pre-equilibrated IVF medium covered with warm mineral oil in a 35 mm culture dish (30 COCs/drop). Spermatozoa (meeting the minimum concentration of 1×10^7^ sperm cells /mL and the sperm motility > 0.6) were added to the drops containing COCs and incubated at 38.5°C in 5% CO_2_ and high humidity for fertilization. COCs were washed and transferred (30 COCs/well) to a Nunc 4-well multi-dish containing 500 µL of embryo culture medium covered with 500 µL mineral oil, 6h (bovine) and 17h (ovine, goat) after insemination, and cultured at 38.5°C in air containing 5% CO_2_ and high humidity for 48 h to assess the cleavage rate.

### Statistical analysis

Data are presented as mean ± SEM and were analyzed using ANOVA to assess differences between sperm before and after cryopreservation. A probability level of *P* < 0.05 was considered significant. The least significant difference test was used for multiple comparisons. All analyses were performed using statistical software SPSS 17.0.

### Animal care

The Institutional Animal Care and Use Committee of Inner Mongolia University approved the experimental protocols used in this study for bovine, ovine, and goat (SYXK2014-0002).

## Results

### Cryopreservation affects acrosome integrity of bovine, ovine, and goat sperm

The acrosome integrity rates of bovine, ovine, and goat sperm before and after cryopreservation were 98.25% vs. 94.00%, 98.50% vs. 89.00%, and 98.15% vs. 88.30%, following Giemsa staning and ultrastructure analysis of treated spermatozoa, respectively (Table 1, Figure1), showing that the rate of sperm acrosome integrity decreased significantly (*P* < 0.01) after cryopreservation. There was no significant difference in the rate of sperm acrosome integrity in bovine, ovine, and goats before cryopreservation (*P* > 0.05). Acrosome integrity of bovine sperm was significantly higher (*P* < 0.01) than that of ovine and goat sperm after cryopreservation.

**Table 1:** Acrosome integrity rates of bovine, ovine, and goat sperm before and after cryopreservation.

Acrosome integrity (%)		
Species	Fresh	Frozen
Bovine	98.25 ± 0.95^AA’^	94.00 ± 0.81^BA’^
Ovine	98.50 ± 0.57^AA’^	89.00 ± 2.87^BB’^
Goat	98.15 ± 1.13^AA’^	88.30 ± 2.32^BB’^

^A, A’^ Indicates differences in the same row (*P* < 0.01); ^B, A’^ Indicates differences in the same column (*P* < 0.01).

### Cryopreservation affects HYD and ACE activity in bovine, ovine and goat sperm

**Figure 2:**
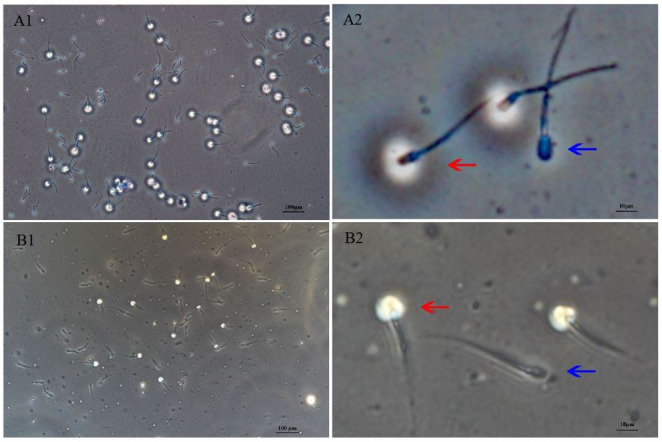
A1 and A2 (part enlarged from A1): A view of HYD treated sperm showed two type of sperm with or without HYD acrosin reaction after cryopreservation sperm in bovine. B1 and B2 (part enlarged from B1): A view of HYD treated sperm showed two type of sperm with or without HYD acrosin reaction after cryopreservation sperm in goat. The red arrow indicates the HYD reaction in sperm with a circular ring, and the blue arrow indicates no HYD reaction in sperm without a circular ring HYD reaction represent sperm mobility.

The percentage of sperm with HYD activity of bovine, ovine, and goat sperm before and after cryopreservation was 84.55% vs. 71.10%, 84.50% vs. 67.60%, and 73.30% vs. 58.52%, respectively (Table 2, Figure 2). The percentage of sperm with HYD activity of bovine (71.10 ± 1.10%) and ovine sperm (67.60 ± 5.56%) was higher (*P* < 0.01) than that of goat sperm (58.52 ± 5.59%). The ACE activity of bovine, ovine and goat sperm before and after cryopreservation was 263.60 μIU/10^6^ vs. 100. 48 μIU/10^6^, 111.10 μIU/10^6^ vs. 57.88 μIU/10^6^, and 95.21 μIU/10^6^ vs. 50.30 μIU/10^6^, respectively (Table 2, Figure 2). The ACE activity of bovine sperm (100.48 ± 13.44 μIU/10^6^) was higher (*P* < 0.01) than that of ovine (57.88 ± 9.58 μIU/10^6^) or goat sperm (50.30 ± 6.17 μIU/10^6^). Overall, HYD and ACE activity of bovine sperm was significantly higher than that of both ovine and goat sperm.

**Table 2:** Effects of cryopreservation on the percentage of sperm with HYD and ACE activity in bovine, ovine, and goat sperm.

HYD activity (%)			ACE activity (μIU/10^6^)	
Species	Fresh	Frozen	Fresh	Frozen
Bovine	84.55 ± 3.25^AA’^	71.10 ± 1.10^BA’^	263.60 ± 28.99^AA’^	100.48 ± 13.44^BA’^
Ovine	84.50 ± 1.77^AA’^	67.60 ± 5.56^BA’^	111.10 ± 23.83^AB’^	57.88 ± 9.58^BB’^
Goat	73.30 ± 4.42^AB’^	58.52 ± 5.59^BB’^	95.21 ± 12.5^AB’^	50.30 ± 6.17^BB’^

^A, A’^ Indicates differences in the same row (*P* < 0.01); ^B, A’^ Indicates differences in the same column (*P* < 0.01).

### Cryopreservation affects motility of bovine, ovine and goat sperm

The motility rates of bovine, ovine, and goat sperm before and after cryopreservation were 68.08% vs. 44.58%, 63.82% vs. 40.29%, 67.69% vs. 38.77% (Table 3). The motility rate of sperm after cryopreservation decreased significantly in all of three species respectively (*P* < 0.01). Therefore, the data demonstrated that the motility rates of bovine and goat sperm were significantly higher than that of ovine sperm before cryopreservation (*P* < 0.01), instead, after cryopreservation the motility rate of bovine sperm was significantly higher than that of both ovine and goat sperm (*P* < 0.01).

**Table 3:** Effects of cryopreservation on bovine, ovine, and goat sperm motility.

Motility (%)		
Species	Fresh	Frozen
Bovine	68.08 ± 8.08^AA’^	44.58 ± 3.96^BA’^
Ovine	63.82 ± 4.51^AB’^	40.29 ± 2.78^BB’^
Goat	67.69 ± 3.30^AA’^	38.77 ± 3.11^BB’^

^A, A’^ Indicates differences in the same row (*P* < 0.01); ^B, A’^ Indicates differences in the same column (*P* < 0.01).

### Cryopreservation affects IVF ability in bovine, ovine and goat sperm

The cleavage rates of eggs fertilized in vitro with bovine, ovine, and goat sperm before and after cryopreservation were 64.2% vs 62.9%, 58.5% vs 49.2%, and 52.1% vs 48.3% respectively (Table 4). Cryopreservation had no significant effect on cleavage rate of bovine sperm (*P* > 0.05); however, the cleavage rates of eggs fertilized with cryopreserved ovine and goat sperm declined significantly (*P* < 0.01). The cleavage rate of eggs fertilized with fresh bovine sperm was significantly higher than that of fresh ovine sperm (*P* < 0.05) and fresh goat sperm (*P* < 0.01). The cleavage rate of eggs fertilized with cryopreserved bovine sperm was significantly higher than that of cryopreserved ovine and goat sperm (*P* < 0.01). The results showed that the cleavage rate of eggs fertilized with fresh bovine sperm was significantly higher than ovine and goat sperm.

**Table 4:** Cleavage rates of eggs fertilized in vitro with bovine, ovine, and goat sperm before and after cryopreservation.

Cleavage rate (cleavage number/number of COCs, %)		
Species	Fresh	Frozen
Bovine	(132/206) 64.2^AA’^	(129/205) 62.9^AA’^
Ovine	(118/202) 58.5^Ac’^	(99/202) 49.2^BB’^
Goat	(103/198) 52.1^AB’^	(96/199) 48.3^BB’^

^A,B’^Indicates differences in the same row (*P* < 0.01); ^A, A’^ Indicates differences in the same column (*P* < 0.01); ^Ac’^ Indicates differences in the same column (*P* < 0.05).

### Correlation analysis between motility rate, acrosome integrity, and the percentage of Sperm with HYD and ACE activity of fresh and frozen bovine, ovine and goat sperm

The motility rates of fresh and frozen bovine, ovine, and goat sperm were positively correlated with the rates of acrosome integrity (bovine: 0.870 vs. 0.816; ovine: 0.905 vs. 0.854; goat: 0.853 vs. 0.870), but this was not significant (Table 5, *P* > 0.05). A significant positive correlation was found between fresh and frozen bovine, ovine and goat sperm motility rates, and HYD and ACE activity (Table 5, *P* < 0.01).

**Table 5:** The correlation between motility rate and acrosome integrity rate, percentage of sperm with HYD activity and ACE activity of bovine, ovine, and goat sperm.

Group	Acrosome integrity^1)^	HYD activity^1)^	ACE activity^1)^
Bovine sperm motility^1^^)^ (fresh)	0.870	0.773**	0.856**
Bovine sperm motility^1)^ (frozen)	0.816	0.782**	0.891**
Ovine sperm motility^1)^ (fresh)	0.905	0.840**	0.814*
Ovine sperm motility^1)^ (frozen)	0.854	0.806*	0.825*
Goat sperm motility^1)^ (fresh)	0.853	0.794**	0.684*
Goat sperm motility^1)^ (frozen)	0.870	0.653*	0.783*

1) Basic correlation data of Calculating obtained from Table 1, Table 2 and Table 3. *P < 0.05, **P < 0.01.

The percentage of sperm with HYD activity in bovine and ovine sperm was significantly higher than that in goat sperm before and after cryopreservation (bovine: 0.773 vs. 0.782; ovine: 0.840 vs. 0.806; goat: 0.794 vs. 0.653; Table 5, *P* < 0.01). And also, the ACE activity of bovine sperm was significantly higher than that of ovine or goat sperm (bovine: 0.856 vs. 0.891; ovine: 0.814 vs. 0.825; goat: 0.684 vs. 0.783; Table 5, *P* < 0.01), which highlighting species differences in the percentage of sperm with HYD and ACE activity in fresh bovine, ovine, and goat sperm. After cryopreservation, the percentage of sperm with HYD and ACE activity in bovine, ovine, and goat sperm was significantly reduced, with the lowest activity in goat sperm and the highest activity in bovine sperm (Table 5, Figure 2). Taken together, HYD and ACE activity were very effective for appraising sperm activity.

## Discussion

Acrosome integrity is known to be a prerequisite for successful fertilization, the morphology of a sperm head, especially the acrosome area, has been related to the ability of sperm to bind zona pellucida (Cui et al., 2000). Previous studies have shown that cryopreservation did not affect the integrity of the inner acrosome membrane, the release process and function of HYD and other bioactive substances in the acrosome, or the ability of sperm to penetrate eggs (Sun et al., 2019). In our study, type A1 and A2 sperm both had intact acrosomes, while type A3 and A4 sperm had broken acrosome membranes with content release, which may affect sperm fertilization ability (Figure 1). the number of intact acrosomes in bovine spermatozoa after cryopreservation was significantly higher than that of ovine and goat sperm, which was correlated with fertilization outcomes in our feild AI applications (unpublished data). Change in lipid composition is a key step in sperm capacitation, bovine sperm had a variety of lipid membrane (Lafleur et al., 2010). Thus, We thought that the lipid membrane structure of bovine sperm may be more resistant to cold stress rather than ovine and goat.

Sperm motility is the most important index to evaluate sperm quality and fertilization ability. The abnormal structure of any part or organelle of sperm can affect sperm motility. The reduction of sperm motility in our study may be due to damage of the plasma membrane and acrosome caused by physical injury, cold stimulation and oxidation after cryopreservation, and a decline of mitochondrial metabolism and energy metabolism. Our results revealed no significant difference in fresh sperm motility among bovine, ovine, and goat, but the motility of frozen bovine sperm was significantly higher than that of frozen ovine or goat sperm (Table 3).

Sperm capacitation need acrosome enzyme, which are closely involved in the reproductive ability of sperm and can be used as an indicator of animal fertility (Stival et al., 2016). HYD, found in the sperm acrosome, is an important enzyme in sperm fertilization (Kurien and Kathiresan, 2012), playing a significant role in the process of sperm penetration. ACE is a proteolytic enzyme in the sperm acrosome, which is involved in the complex fertilization process, and its activity can directly affect fertility (Zoppino et al., 2012). Enzymes are important indicators of sperm quality (Howes and Jones, 2002). During sperm cryopreservation, a series of changes occur in the sperm plasma membrane and sperm head ultrastructure, resulting in the loss of enzyme contents.

Our study showed that the motility of bovine, ovine, and goat sperm was positively correlated with the percentage of sperm HYD and ACE activity (Table 5, *P* < 0.01). IVF is a reliable approach to assess sperm fertilization ability (Table 4). When spermatozoa are stored at low temperatures for long periods of time, their enzyme activity, motility and fertilization rates are significantly reduced (Chen et al., 2010). In the present study, the fertilization rate of bovine sperm before and after cryopreservation was significantly higher than that of ovine and goat sperm (Table 4, *P* < 0.01) . Correspondingly, acrosome integrity, sperm motility, and the percentage of sperm HYD and ACE activity of bovine sperm were higher than in ovine and goat sperm, both before and after cryopreservation, revealing a higher resistance of bovine sperm to freezing than ovine and goat sperm.

## Conclusion

In conclusion, cryopreservation is a indispensable process for the genetic expanding of domestic animal semen, but also it is a negative effect on bovine, ovine, and goat sperm acrosome integrity, motility, and enzyme activity of HYD and ACE. The fertilization ability of ovine and goat sperm significantly decreased after cryopreservation. However, frozen sperm had similar fertilization ability to fresh sperm in bovine. There was no significant difference between ovine and goat sperm indices, except for percentage of sperm with HYD activity. Those points take together, which suggest that more investigations needed for ovine and goat semen cryopreservation to improve theirs AI field applications in the future studies.
